# Endovascular stroke treatment in a small‐volume stroke center

**DOI:** 10.1002/brb3.642

**Published:** 2017-02-28

**Authors:** Gry N. Behzadi, Lars Fjetland, Rajiv Advani, Martin W. Kurz, Kathinka D. Kurz

**Affiliations:** ^1^ Radiological Research GroupDepartment of RadiologyStavanger University HospitalStavangerNorway; ^2^ Neuroscience Research GroupDepartment of NeurologyStavanger University HospitalStavangerNorway; ^3^ Stavanger UniversityStavangerNorway

**Keywords:** endovascular treatment, general interventional radiologists, ischemic stroke, large vessel occlusions, stroke centers

## Abstract

**Introduction:**

Our purpose was to evaluate the safety and efficacy of endovascular treatment (EVT) of stroke caused by large vessel occlusions (LVO) performed by general interventional radiologists in cooperation with stroke neurologists and neuroradiologists at a center with a limited annual number of procedures. We aimed to compare our results with those previously reported from larger stroke centers.

**Patients and Methods:**

A total of 108 patients with acute stroke due to LVO treated with EVT were included. Outcome was measured using the modified Rankin scale (mRS) at 90 days. Efficacy was classified according to the modified thrombolysis in cerebral infarction (mTICI) scoring system. Safety was evaluated according to the incidence of procedural complications and symptomatic intracranial hemorrhage (sICH).

**Results:**

Mean age of the patients was 67.5 years. The median National Institutes of Health Stroke Scale (NIHSS) on hospital admission was 17. Successful revascularization was achieved in 76%. 39.4% experienced a good clinical outcome (mRS<3). Intraprocedural complications were seen in 7.4%. 7.4% suffered a sICH. 21.3% died within 3 months after EVT.

**Discussion:**

The use of general interventional radiologists in EVT of LVO may be a possible approach for improving EVT coverage where availability of specialized neurointerventionalists is challenging. EVT for LVO stroke performed by general interventional radiologists in close cooperation with diagnostic neuroradiologists and stroke neurologists can be safe and efficacious despite the low number of annual procedures.

## 1 Introduction

Stroke is one of the leading causes of morbidity and mortality worldwide. It is estimated that around 87% of all strokes are ischemic in origin (Rosamond et al., 2008). Intravenous thrombolysis (IVT) was previously the gold standard treatment for acute ischemic stroke (Hacke et al., 2008; Tissue plasminogen activator for acute ischemic stroke, 1995). However, in patients with large vessel occlusion (LVO) stroke, endovascular treatment (EVT) has shown to improve clinical outcome (Berkhemer et al., 2015; Campbell et al., 2015; Ding, 2015; Goyal et al., 2015, 2016; Jovin et al., 2015; Pereira et al., 2013; Saver et al., 2015). Subsequently, EVT, in addition to IVT or alone if contraindications for IVT, is now recommended as the treatment of choice in LVO stroke (Jauch et al., 2013; Powers et al., 2015; Wahlgren, 2014).

There is an ongoing discussion about these new recommendations and the implications on the organization of acute stroke care (Tatlisumak, 2015). EVT is preferably performed by specialized interventional neuroradiologists; however, in some countries as in Norway, there are too few of these subspecialists to provide adequate nationwide coverage (Demaerschalk, Raman, Ernstrom, & Meyer, 2012; Pérez de la Ossa et al., 2016; Tatlisumak, 2015).

Time to recanalization is crucial for clinical outcome (Rha & Saver, 2007; Vergouwen et al., 2012), and the transport time to centers offering EVT is a challenge in smaller cities and rural areas and as a result, EVT might not be a realistic and viable option for patients from these areas (Pérez de la Ossa et al., 2016).

It is also assumed that a high number of EVT procedures are needed to achieve the desired technical and clinical results (Connors et al., 2005; Lavine SD et al., 2016; White et al., 2017). Our results from previous studies have suggested that a multidisciplinary approach with close cooperation between general interventional radiologists, stroke neurologists, and diagnostic neuroradiologists might be a way to compensate for the lack of specialized interventional neuroradiologists (Fjetland, et al., 2012). Our center, Stavanger University Hospital, Norway, is a university hospital serving a population of 350,000 inhabitants, is the sole hospital in the region treating acute stroke, and has performed around 20 EVT procedures annually with a total of 108 so far. We reanalyze the safety and efficacy of this approach in order to evaluate its feasibility and potential as a model for other centers.

## 2 Patients and Methods

### 2.1 Patients

From December 2009 to August 2015, all patients with LVO in either the anterior or the posterior circulation treated with EVT were included in the study. EVT was initiated within 6 hours from symptom onset in 96 of the patients. Twelve of the patients had a wake‐up stroke or an unclear onset of stroke. Eligible patients had an arterial occlusion in the internal carotid artery (ICA), the M1 or proximal M2 segment of the middle cerebral artery (MCA), an occlusion of the distal vertebral artery (V4) segment, or basilar artery (BA). Prior to treatment, a CT examination including an unenhanced computed tomography (CT), a perfusion CT (CTP), and a CT angiography (CTA) was performed in most patients. A magnetic resonance imaging (MR) was performed in patients with unknown onset of symptoms or as a supplement to CT in decision making in difficult cases. The MR protocol included diffusion‐weighted images (DWI), T2‐weighted images, fluid‐attenuated inversion recovery (FLAIR) images, T2*‐weighted images, and a time‐of‐flight (TOF) MR angiography of the intracerebral arteries. Patients with intracranial hemorrhage or with a distinct demarcation of an infarction greater than one‐third of the vessel territory on unenhanced CT or DWI‐MR images were excluded from the study. In the posterior circulation, exclusion criteria were massive infarct demarcations in either CT or MR. All patients were older than 18 years of age without any strict upper limit. Patients eligible for IVT were pretreated with intravenous recombinant tissue plasminogen activator (rt‐PA, Actilyse) and transported with ongoing IVT infusion (direct bridging concept) to the angiography suite. Patients arriving too late or having contraindications for IVT were transported directly to the angiography suite after the initial radiological examinations. The decision to treat with EVT was made by a team comprising a stroke neurologist, a general vascular interventional radiologist, and a diagnostic neuroradiologist. This decision was based on both clinical criteria and results of the initial CT or MR examination.

### 2.2 Revascularization procedures

EVT was performed by six experienced general vascular interventional radiologists, working together in teams of two. All procedures were performed in a monoplane angiography suite equipped with a Siemens Artis zee (December 2009‐June 2014) or a Philips Allura FD20 Clarity (Philips, Best; Netherlands) (June 2014‐present moment). After performing a common femoral arterial puncture, the distal tip of a 6‐F guiding sheath was placed in the proximal part of ICA in the anterior circulation and in the subclavian artery in the posterior circulation and an aspiration catheter advanced in an coaxial manner as close as possible to the proximal part of the occluding thrombus. Table 1 offers an overview over the equipment used during the EVT procedures.

**Table 1:** Equipment used by the reperfusion procedure

	Anterior circulation	Posterior circulation
Mechanical thrombectomy with aspiration		
Microcatheter	Prowler Select +^a^	Prowler Select +^a^
Guidewire 0,035	Glidewire^b^	Glidewire^b^
	Advantage^b^	Advantage^b^
Microguidewire	Fathom‐016^c^	Fathom‐016^c^
	Journey^c^	Journey^c^
Guiding catheter	Neuron 6F^d^	Neuron 6F^d^
	Destination 6F^b^	Destination 6F^b^
	ENVOY^a^6F	
	Strada Carotid Guiding Sheath^e^	
Aspiration catheter	Penumbra Reperfusion Catheter 041^d^with Penumbra Separator 041^d^	Penumbra Reperfusion Catheter 041^d^with Penumbra Separator 041^d^
	Penumbra Reperfusion Catheter 032^d^with Penumbra Separator 032^d^	Penumbra Reperfusion Catheter 032^d^with Penumbra Separator 032^d^
	Penumbra Reperfusion Catheter 026^d^with Penumbra Separator 026^d^	Penumbra Reperfusion Catheter 026^d^with Penumbra Separator 026^d^
Mechanical thrombectomy with stent retriever		
Microcatheter	Rebar^f^	Rebar^f^
Microguidewire	Fathom‐016^c^	Fathom‐016^c^
	Journey^c^	Journey^c^
Guiding catheter	Destination 6F^b^	Destination 6F^b^
	Neuron 6F d	Neuron 6F d
	Strada Carotid Guiding Sheath^e^	
Aspiration catheter	Penumbra ACE 64^d^	Penumbra ACE 64^d^
	Penumbra 5 MAX^d^	Penumbra 5 MAX^d^
Stent retriever	Solitaire AB/FR^f^	Solitaire AB/FR^f^
Stents and balloons		
Carotic stent	Adapt carotid stent 4‐9mm^c^	
Intracranial stent	Wingspan 2.5‐4.5mm^c^	Wingspan 2.5‐4.5mm^c^
Balloon	Gateway^c^	Gateway^c^

a Codman & Shurtleff, Inc. Raynham, MA, USA.

b Terumo Medical Corporation, Somerset, NJ USA.

c Boston Scientific Corporation, Natick, MA, USA.

d Penumbra Inc. Alameda, CA, USA.

e St. Jude Medical Inc. St. Paul, MN, USA.

f Microtherapeutics, ev3, Irvine, CA, USA.

From December 2009 up to August 2010, thrombectomy with aspiration using the Penumbra System was the standard reperfusion procedure. From August 2010 onward, the Solitaire FR thrombectomy system combined with the Penumbra Reperfusion Catheter for distal aspiration has been the first treatment of choice.

Acute ICA occlusions were treated with the Adapt carotid stent and intracranial stenosis with the Wingspan stent. The femoral artery puncture site was sealed with FemoSeal at the end of the procedure. Most of the procedures in the first years of the study were performed under general anesthesia. After updated knowledge, we have changed this procedure toward conscious sedation (Powers et al., 2015; Wahlgren, 2014).

### 2.3 Efficacy and safety evaluation

All angiograms were classified by one general interventionalist and two diagnostic neuroradiologists in consensus, who were blinded to clinical data. Efficacy was classified according to the modified thrombolysis in cerebral infarction (mTICI) scoring system (Zaidat et al., 2013). Successful recanalization was defined as mTICI grade 2b or 3 (Zaidat et al., 2013). A follow‐up MR was performed 24 hours after onset in most cases, with the remainder undergoing a CT scan, to determine the infarcted area as well as any complications. Intracranial hemorrhages (ICH) were classified according to the European Cooperative Acute Stroke Study II (ECASS II) (Hacke et al., 1998). Symptomatic ICH (sICH) was defined as ICH associated with a greater than four‐point deterioration of the National Institutes of Health Stroke Scale (NIHSS) score. A supplementary CT or MR examination was performed in cases of clinical deterioration.

Safety of treatment was evaluated according to the incidence of procedural complications (within 24 hr). This was based on findings from angiograms during the procedure, MR or CT scans after the procedure, and clinical–neurologic examinations. Procedure‐related complications included vessel perforation, intramural arterial dissection, emboli into new vascular territories outside that of the target vessel (ENT), and local complications of groin puncture.

### 2.4 Clinical evaluation

All patients underwent clinical assessment including NIHSS scoring performed on admission, the first day after intervention, and on the day of discharge. Modified Rankin scale (mRS) was assessed at three‐month follow‐up by a certificated stroke nurse. Good neurologic outcome was defined as a mRS score 0–2 at 3 months, and poor neurologic outcome was defined as a mRS score 3–6.

### 2.5 Statistics

All statistical analyses were performed using SPSS Statistics version 21 (IBM Cooperation, Armonk, NY, USA). Baseline variables and variable changes were examined using one‐way analysis of variance (ANOVA) and Pearson's chi‐squared test as appropriate.

### 2.6 Informed consent

Informed consent was obtained from either the patient or a legal representative before treatment.

### 2.7 Ethical approval

All procedures performed in studies involving human participants were in accordance with the ethical standards of the institutional and/or national research committee and with the 1964 Helsinki Declaration and its later amendments or comparable ethical standards. This study was approved by the regional ethical committee.

## 3 Results

Of 2906 patients admitted with acute ischemic stroke, 108 patients (3.7%) were treated with EVT during the study period. Clinical patient baseline characteristics including their cardiovascular risk factors are shown in Table 2; 92 patients (85.2%) had an occlusion in the anterior circulation and 16 patients (14.8%) in the posterior circulation. The site of the occlusions is shown in Table 3. During the period, different EVT devices were used (Table 1).

**Table 2:** Patient baseline characteristics and cardiovascular risk factors

Mean years of age (range)	67.5 (31–92)
Female gender (%)	42 (38.9)
Cardiovascular burden (%)	
Hypertension	61 (56.5)
Diabetes mellitus	12 (11.1)
Previous cardiovascular events	14 (13.0)
Previous cerebral vascular events	14 (13. 0)

**Table 3:** Site of occlusion and clinical outcome at 90 days

	*n* (%)	mRS 0–2 (%)
ICA^a^	4 (3.7)	0
Carotid T	10 (9.3)	4 (40)
Tandem lesions^b^	23 (21.3)	9 (39.1)
MCA^c^	55 (50.9)	23 (41.8)
Basilar artery	16 (14.8)	7 (43.8)

a Internal carotid artery.

b Tandem lesions: ICA and MCA.

c MCA: middle cerebral artery.

Twelve patients had a preceding extracranial stenosis or occlusion in the ICA and were acute‐stented. Additionally, three patients were treated with a stent in the BA, one in the VA, and one in the MCA; 69 (63.9%) of the patients received IVT prior to EVT. Median time from onset to reperfusion was 241 min in patients with known onset.

### 3.1 Revascularization

All 108 patients had an initial mTICI score of 0 or 1. In a total of 82 patients (76%), an angiographically good result could be seen after EVT (Table 4). An mTICI score of 2b or 3 was seen in 72 patients (78.3%) with occlusions in the anterior circulation and 10 patients (62.5%) with occlusions in the posterior circulation.

**Table 4:** Angiographic result by mTICI score

Angiographic result	Total (*n* = 108) (%)	Anterior circulation (*n* = 92) (%)	Posterior circulation (*n* = 16) (%)
mTICI 0	8 (7.4)	4 (4.4)	4 (25)
mTICI 1	8 (7.4)	7 (7.6)	1 (6.3)
mTICI 2a	10 (9.3)	9 (9.8)	1 (6.3)
mTICI 2b	39 (36.1)	37 (40.2)	2 (12.5)
mTICI 3	43 (39.8)	35 (38.0)	8 (50)

### 3.2 Safety

Intraprocedural complications were seen in eight patients (7.4%): two extravasations (self‐limiting seen only during the EVT procedures), one perforation, two AV fistulas, two losses of catheter material, and one artery dissection. One of the AV fistulas occurred in a patient with an MCA occlusion who developed a carotid cavernous fistula (CCF) during the EVT. It is unsure whether this fistula was preexisting or iatrogenic. This patient was closely followed up both clinically and radiologically and remained asymptomatic.

The other AV fistula was seen only under the procedure and was self‐limiting. It could not be seen at the end of the procedure.

ENT was seen in three patients in the anterior circulation (3.3%). These emboli were all located in the anterior cerebral artery (ACA) branches and were related to challenging interventional procedures in the MCA. ICH was found in 37 patients (34.3%): 20 patients (18.5%) showed an HI1 or HI2, and 17 patients (15.7%) a P1 or P2. Eight patients (7.4%) suffered a sICH; all of them occurred in patients with LVO in the anterior circulation (8.6%). There were significantly more ICH in patients not successfully recanalized (*p* = .04).

The patient outcomes and complications are shown in Table 5.

**Table 5:** Patient outcomes and complications

	NIHSS before treatment a	NIHSS after treatment^a^	NIHSS on discharge^a^	mRS^a^^,^ b	Mortality^b^	sICH	Intraprocedural complications
All patients	17 (105)	13 (100)	6 (84)	3.5 (108)	23 (108) 21.3%	8 (108) 7.4%	8 (108) 7.4%
mTICI 2b‐3	17.5 (82)	9.5 (82)	5 (77)	2 (75)	8 (82) 9.8%	3 (82) 3.7%	4 (82) 4.9%
mTICI 0‐2a	17 (23)	20 (19)	15.5 (10)	6 (23)	15 (23) 65.2%	5 (23) 21.7%	4 (23) 17.4%
*p*‐value	.97	<.001	<.001	<.001	<.001	.04	.33

a Median.

b 3 months.

() number of patients.

### 3.3 Clinical outcome

Forty‐three patients (39.8%) in the entire cohort experienced a good clinical outcome (mRS < 3). The clinical outcomes in relation to the site of occlusion are shown in Table 3.

In addition, nine patients (8, 3%) had an mRS of 3 after 3 months.

The median NIHSS scores and the 90‐day mRS are displayed according to recanalization status in Table 5. Those successfully recanalized showed a highly significant improvement in outcome, a better functional outcome at three months, and a significantly lower mortality rate.

### 3.4 Mortality

Twenty‐three patients (21.3%) died within 3 months after EVT. Of those successfully recanalized, eight patients (9.8%) died, two of them as a direct result of the infarction. The six patients successfully recanalized that died but not from the initial infarction, died of ischemic heart disease or pneumonia. One patient died of bleeding from a previously unrecognized rectal tumor.

Fifteen of those (65.2%) not successfully recanalized died; 14 of them (93.3%) as a direct result of the infarction. Sixteen of the 92 patients (17.4%) with an occlusion in the anterior circulation died (in patients < 80 years 9 of 59 patients (13.2%) and only two with an isolated MCA occlusion (5.6%)). Six of the 16 patients (37.5%) with an occlusion in the posterior circulation died (in patients <80 years four of nine patients (30.8%)).

## 4 Discussion

In this study, we can show that EVT of LVO stroke can be performed by general interventional radiologists in close cooperation with diagnostic neuroradiologists and stroke neurologists. Despite the limited number of procedures performed annually, this approach seems to be safe and efficacious. From 2009 onward, we have performed around 20 EVT procedures annually and in total treated more than 100 patients. As we lack interventional neuroradiologists, our approach may be capable to compensate for this disadvantage. Patients with LVO treated with EVT performed by general interventional radiologists may get an outcome far better than alternative IVT treatment, and literally our results are comparable to those from high‐volume stroke centers where neurointerventional specialists perform EVT (Berkhemer et al., 2015; Campbell et al., 2015; Goyal et al., 2015; Jovin et al., 2015; Saver et al., 2015). During our treatments, there are always two interventional radiologists performing the EVT as a team. This results in more procedure experience for each of the interventionists.

In the anterior circulation, a good result angiographically with an mTICI score of 2b or 3 was achieved in 78.3%. This result is in line with five published randomized controlled trials (RCTs) from larger neurointerventional centers reporting successful reopening rates of 58.7–88% (Berkhemer et al., 2015; Campbell et al., 2015; Goyal et al., 2015; Jovin et al., 2015; Saver et al., 2015). Only the EXTEND‐IA and the SWIFT PRIME studies (35 and 98 patients) reported higher successful recanalization rates of 86.2% and 88.0%, respectively (Campbell et al., 2015; Saver et al., 2015). However, in both these studies, the patients were highly selected with several inclusion criteria including an upper age limit of 80 years. Additionally, all the patients in these two studies were treated using stent retrievers exclusively. When comparing “real life data” from the MR CLEAN, ESCAPE, and REVASCAT studies (Berkhemer et al., 2015; Goyal et al., 2015; Jovin et al., 2015), the contributing centers achieved a revascularization rates of between 58.7% and 72.4%.

In EJMINT, training guidelines for EVT were published in 2016 and the minimum suggestions for successful recanalization are mTICI 2b or 3 in at least 60% of cases (Lavine SD et al., 2016). Procedural complications were seen in 7.4% of the patients, in line with current literature (Behme et al., 2014; Campbell et al., 2015; Goyal et al., 2015; Jovin et al., 2015; Pereira et al., 2013; Saver et al., 2015). sICH was observed in 7.4% of the patients. In the study from Behme et al. (Behme et al., 2014), sICH was seen in 5%. The EJMINT guidelines recommend a rate less than 10% (Lavine SD et al., 2016). ENT was seen in three patients (2.8%), all of them suffering a poor clinical outcome. This serious complication has previously been reported to be between 4.9% and 6% in published RCTs (Berkhemer et al., 2015; Campbell et al., 2015; Jovin et al., 2015). In the EJMINT guidelines, the recommendation is ENT less than 15% (Lavine SD et al., 2016).

The significance of revascularization is emphasized by the correlation of successful recanalization with good clinical outcome in our cohort. Those successfully recanalized exhibited an average reduction in NIHSS of 11 as opposed to 1.5 in those not recanalized (Table 5). Of those not successfully recanalized, not a single patient achieved a mRS <4 at 90 days and the median mRS was 6. This is in stark contrast to those where successful recanalization was achieved having a median mRS of 2 at 90 days. In our patient cohort, 17.4% of the patients with an occlusion in the anterior circulation died. This is similar to the 18.9% and 18.4% reported by the MR CLEAN and REVASCAT studies, respectively. In the posterior circulation cohort, 37.5% of the patients died. This is comparable to the mortality rate of 35% in the ENDOSTROKE study where 148 patients with basilar occlusions were treated with EVT (Singer et al., 2015).

We had no upper age limit in our patient cohort. Patients ≥80 years old are known to have poorer outcomes after suffering a stroke (Castonguay et al., 2014; Ford et al., 2010; Kurre et al., 2013). Subsequently, only 19.2% of these patients exhibited a good outcome, while 34.6% of them died within three months after the stroke. In our cohort, those <80 years of age with an isolated occlusion of the MCA had a mortality rate of only 5.6%.

In the subgroup of patients with a basilar artery occlusion, 37.5% of the patients died, while 43.8% had a good outcome at 90 days. The natural course of basilar artery occlusions is known to be disastrous with mortality rates as high as 90% (Sairanen et al., 2011), yet by achieving successful recanalization, the functional outcome is often satisfactory (Ottomeyer et al., 2012; Sairanen et al., 2011; Singer et al., 2015; Vergouwen et al., 2012; Yoon et al., 2015). In the ENDOSTROKE study, 34% had a good outcome and 35% died (Singer et al., 2015). Our results reflect the importance of attempting recanalization in the posterior circulation.

The main weakness of our cohort study is the variation in the interventional devices used. This is due to technical developments in the endovascular field during the study period. Yet, our study is a “real life” observational study of EVT including the entire adult population in our catchment area. The prospective inclusion of all the patients and complete patient follow‐up are undisputable strengths of our study. We have clearly demonstrated that EVT for LVO stroke performed by general interventional radiologists in close cooperation with diagnostic neuroradiologists and stroke neurologists can be safe and efficacious. Even though the number of procedures performed annually is quite low, we have achieved high revascularization rates, low complication rates, and most importantly good patient outcomes. Given the superiority of EVT as compared to IVT alone for LVO stroke, it should ideally be available to the entire population. The lack of neurointerventional specialists in several European countries makes it almost impossible to provide adequate nationwide coverage, leading to the subsequent lack of a vital therapeutic modality for a large group of patients. Transportation to high‐volume stroke centers is often time‐consuming. As a time delay of 15 minutes may lead to a 10% reduction in chance of good outcome, time is essential in the context of EVT (Saver et al., 2016). Thus, EVT performed in smaller hospitals might be a better alternative than time‐consuming transfer to larger hospitals. To ensure good quality and safety of EVT in small‐volume centers, a quality register should be established, in line with high‐volume centers. A multidisciplinary approach using general interventional radiologists as part of the team may be a viable model for improving EVT coverage in areas with a geographical situation where reaching stroke centers with specialized neurointerventionalists in time is challenging.

## Conflict of Interest

The authors declare that there are no conflicts of interest.
